# A High-Sensitivity U-Shaped Optical Fiber SPR Sensor Based on ITO Coating

**DOI:** 10.3390/s25133911

**Published:** 2025-06-23

**Authors:** Chuhan Ye, Zhibo Li, Wenhao Kang, Lei Hou

**Affiliations:** 1 College of Physics and Electronic Engineering, Hainan Normal University, Haikou 571158, China; 202313085408010@hainnu.edu.cn (C.Y.); lizhibo@hainnu.edu.cn (Z.L.); 2 Department of Industrial and Systems Engineering, The Hong Kong Polytechnic University, Hong Kong 999077, China; 3 Research Center for Frontier Fundamental Studies, Zhejiang Lab, Hangzhou 311100, China

**Keywords:** refractive index, U-shaped sensor, indium tin oxide, surface plasmon resonance

## Abstract

This paper proposes a high-sensitivity U-shaped optical fiber sensor based on indium tin oxide (ITO) for surface plasmon resonance (SPR) sensing. Finite element simulations reveal that introducing ITO enhances the surface electric field strength by 1.15× compared to conventional designs, directly boosting sensitivity. The U-shaped structure optimizes evanescent wave–metal film interaction, further improving performance. In an external refractive index (RI) range of 1.334–1.374 RIU, the sensor achieves a sensitivity of 4333 nm/RIU (1.85× higher than traditional fiber sensors) and a figure of merit (FOM) of 21.7 RIU^−1^ (1.68× improvement). Repeatability tests show a low relative standard deviation (RSD) of 0.4236% for RI measurements, with a maximum error of 0.00018 RIU, confirming excellent stability. The ITO coating’s strong adhesion ensures long-term reliability. With its simple structure, ease of fabrication, and superior sensitivity/FOM, this SPR sensor is well-suited for high-precision biochemical detection in intelligent sensing systems.

## 1. Introduction

Optical fiber SPR sensors have developed rapidly in recent years due to their compact size, flexible structure, easy operation, and low cost. In 1993, Jorgenson and Yee successfully developed the first optical fiber SPR sensor by combining optical fiber technology with SPR technology for the first time [1]. Such sensors show high sensitivity to small changes in the RI of the environment, as well as good biocompatibility and the ability to selectively absorb light at specific wavelengths, and thus have been widely used in many fields, including in the detection of a solution RI [2], the detection of IgG biological proteins [3], the detection of heavy metal ions [4], the detection of mi-RNAs [5], the detection of urea concentration [6], etc. However, with increasing research on optical fiber SPR sensors, the question of how to achieve higher sensitivity in the trace detection of small-molecule biomarkers has become a key issue in this research field [7,8].

Currently, researchers mainly focus on the two dimensions of substrate structure optimization and novel material application to enhance the sensitivity of optical fiber SPR sensors. On the one hand, with the continuous advancement of modern preparation techniques, a variety of innovative optical fiber substrate structures have emerged, such as the U-shaped structure [9], the S-taper structure [10], and the side-polished D-shaped structure [11]. These structural designs significantly enhance the performance of the sensors by changing the way light interacts with the sensing region. In the U-shaped structure, for example, the bending region is designed with a gradually decreasing angle of incidence. This causes the total quantity of reflections at every length of the material to increase. Swift wave (SW) leakage in the bending zone is more significant and intense as a result of this design, with greater penetration depth, allowing for increased interaction between SWs and the external metal film. Consequently, this configuration significantly boosts the sensor’s RI sensitivity. On the other hand, the emergence of new materials also provides strong support for improvement in sensor sensitivity. Graphene, as a two-dimensional material with excellent physicochemical properties, was first applied to optical fiber SPR sensors after significantly improving the sensitivity of the sensors [12]. Inspired by this, numerous low-dimensional materials have been emerging, such as emerging metal oxides and black phosphorus. These materials further enhance the performance of sensors by enhancing the localization effect of the electromagnetic field, providing a new impetus for the rapid development of SPR sensors.

ITO is a replacement solid solution that typically consists of about 90% indium oxide (In_2_O_3_) and 10% stannic oxide (SnO_2_). This material appears as a transparent, tea-colored film or yellowish-gray lump with excellent properties such as high conductivity, a high dielectric constant, strong light confinement, a high RI, and resistance to corrosion by a variety of electrolyte media. These properties make ITO an important tool in the field of SPR sensors. In research on SPR sensors, ITO has been successfully applied to enhance sensor performance. For example, Woyessa et al. proposed a cladding-etched multimode polymer optical fiber-based SPR sensor coated with a 40 nm thickness Au film and a 25 nm thickness ITO film with a sensitivity of up to 2258 nm/RIU [13]. In addition, Mishra et al. theoretically designed an SPR sensing probe based on fiber coupling with ITO thin films, which exhibited extremely high sensitivity in the infrared band [14]. These studies have shown that ITO thin films can significantly enhance the performance of SPR sensors, especially in improving sensitivity.

Building upon current research in optical fiber SPR sensors, this study presents dual optimizations in both material composition and structural design. A novel sensitization strategy is proposed by coating a conventional SPR optical fiber with an indium tin oxide (ITO) film on its outer surface. COMSOL Multiphysics (version 6.2) simulations reveal that depositing a 70 nm ITO layer atop a 50 nm Au film enhances the electric field (EF) intensity by a factor of 1.15 compared to a pure Au film configuration, demonstrating that ITO integration significantly amplifies SPR excitation. Furthermore, a U-shaped multimode–single-mode–multimode (MSM) fiber structure is designed to further improve sensor performance. Through this combined material–structural optimization, the sensor achieves a sensitivity of 4333 nm/RIU within a refractive index (RI) range of 1.334–1.374, representing an 85% enhancement over conventional fiber-optic SPR sensors. The significant improvement in sensor performance is verified by numerical simulation and RI measurement experiments, indicating that the optimization scheme can effectively improve the performance of the optical fiber sensor for SPR and provide a new technological method for high-sensitivity detection.

## 2. Theoretical Analysis

### 2.1. Sensor Modeling

**Figure 1:**
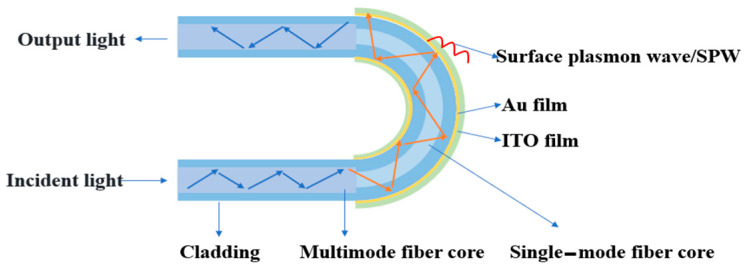
Sensor structure model.

A U-shaped optical fiber sensing structure with Au/ITO film layers was designed, utilizing a multimode fiber–single-mode fiber–multimode fiber (MMF-SMF-MMF) configuration to excite surface plasmon resonance (SPR), as illustrated in Figure 1. The sensing region had a defined length of 1 cm. Owing to the core diameter mismatch between the single-mode fiber (SMF, 9.0 μm core) and multimode fiber (MMF, 62.5 μm core), light leakage into the cladding occurs during propagation. This leaked light interacts with free electrons in the Au/ITO film layers, thereby inducing SPR excitation. The high carrier concentration of ITO enhances the strength of the electronic oscillations, thereby increasing the sensitivity of the SPR signal.

### 2.2. Sensor Mechanism Analysis

Surface plasmon resonance (SPR) optical fiber sensors represent a novel class of high-sensitivity biosensors based on the excitation of surface plasmon waves (SPWs) at metal–dielectric interfaces. The sensing mechanism relies on a thin metal film (e.g., Au or Ag) coated on a fiber core, where incident light couples with free electrons under phase-matching conditions (kx = ksp), inducing collective electron oscillations and SPW propagation. This coupling causes a sharp attenuation in transmitted light intensity, manifesting as a characteristic absorption dip in the spectrum.

The resonance wavelength (RW) is highly sensitive to the refractive index (RI) of the adjacent dielectric medium. Minute RI variations (e.g., RIU) alter SPW propagation constants, shifting the RW proportionally. Thus, by precisely monitoring RW shifts, the sensor enables the highly accurate detection of environmental changes, such as RI variations.

Refractive index (RI) measurement serves as a direct method to quantify the sensitivity of surface plasmon resonance (SPR) sensors. Sensitivity (S) is defined as the resonance wavelength shift (Δλ) per unit change in the analyte’s refractive index (Δn), expressed as

(1) $$S=\frac{\Delta \lambda }{\Delta n}$$

where the sensitivity is S with units of nm/RIU. The full width at half maximum (FWHM) of the SPR transmission spectrum, measured as the wavelength interval between the midpoints of the resonance dip, critically influences sensor resolution. To holistically evaluate performance, the figure of merit (FOM) is introduced as the ratio of sensitivity to FWHM:

(2) $$FOM=\frac{S}{FWHM}$$

### 2.3. Field Enhancement by ITO

It is known that the enhancement of the EF at the sensing surface leads to an expansion of its region of action, i.e., an increase in the volume of action of the swift field at the sensing interface. This change can significantly enhance the SPR phenomenon and, thus, the sensitivity of the sensor. A comparative analysis of the strength of the EFs on the surfaces of the two sensor structures and the area of their action allows an assessment of the effect of the sensitivity enhancement. The simulation results show that the EF strength of the optical fiber/Au/ITO composite film layer structure is significantly enhanced, and the EF action region is further broadened. As a result, the structure can effectively enhance the SPR phenomenon and thus significantly improve the sensitivity of the sensor.

In this paper, based on the relationship between the SPR phenomenon and EF enhancement, optical fiber SPR sensors are optimized from both theoretical and experimental perspectives. It has been shown that the SPR phenomenon can significantly enhance the EF strength on the surface of the sensor, and the sensitivity of the SPR phenomenon can also be further enhanced by enhancing the EF strength [15]. In this study, the EF strengths of the two sensor structures were calculated by simulation software. The RI of the ambient medium was set to 1.33, with the 2D intercepts drawn at the same position in the 2D simulation models of the two sensing structures, respectively, and the values of the EF on the intercepts were calculated.

**Figure 2:**
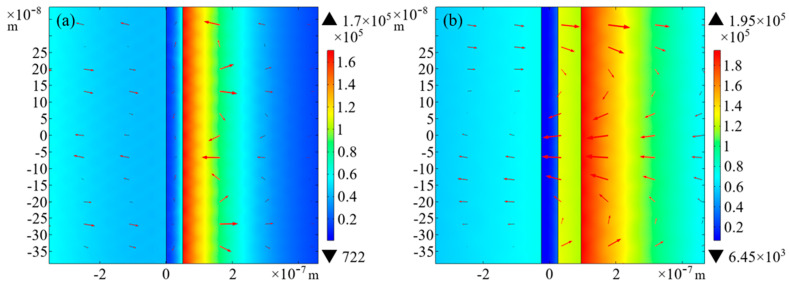
Images of electric field strength distribution. (**a**) Au film structure. (**b**) Au/ITO film layer structure.

Figure 2 illustrates the electric field (EF) intensity distribution, revealing enhanced field confinement at the Au/ITO interface compared to the pure Au structure. The results show that at the wavelength where the SPR phenomenon occurs, the EF strength of the Au film of the optical fiber/Au film SPR sensing structure on the surface in contact with the external ambient medium is 1.78 × 10^5^ V/m, whereas the EF strength of the optical fiber/Au/ITO composite film layer sensing structure under the same conditions increases to 1.95 × 10^5^ V/m. This is due to the fact that the high carrier concentration of ITO enhances the strength of the electronic oscillations, thereby increasing the EF strength. This indicates that the introduction of ITO film significantly enhances the EF strength in the SPR phenomenon, thus providing strong support for enhancing sensor performance.

### 2.4. Simulation Analysis of Sensor Structural Parameters

In terms of the choice of material for the metal layer of the SPR sensor, current research usually favors the use of the precious metals Au or Ag to excite the SPR phenomenon. Ag, as a precious metal, has high reflectance in the visible and near-infrared bands, enabling high sensitivity, small full widths at half maximum (FWHMs), and good resolution, resulting in excellent performance in SPR sensors. However, Ag is highly susceptible to oxidation in air, which limits its stability in practical applications. In contrast, the sensor with the Au film structure, although slightly lower in detection sensitivity than the Ag film structure, is significantly more chemically stable than the Ag film and is easier to implement in experiments. Therefore, most scholars prefer to choose Au as the precious metal nanofilm layer to stimulate the SPR phenomenon in practical applications [16]. In this study, based on the above analysis, Au was also chosen as the material for the metal layer of the sensor structure to ensure that the sensor has good chemical stability along with high sensitivity.

**Figure 3:**
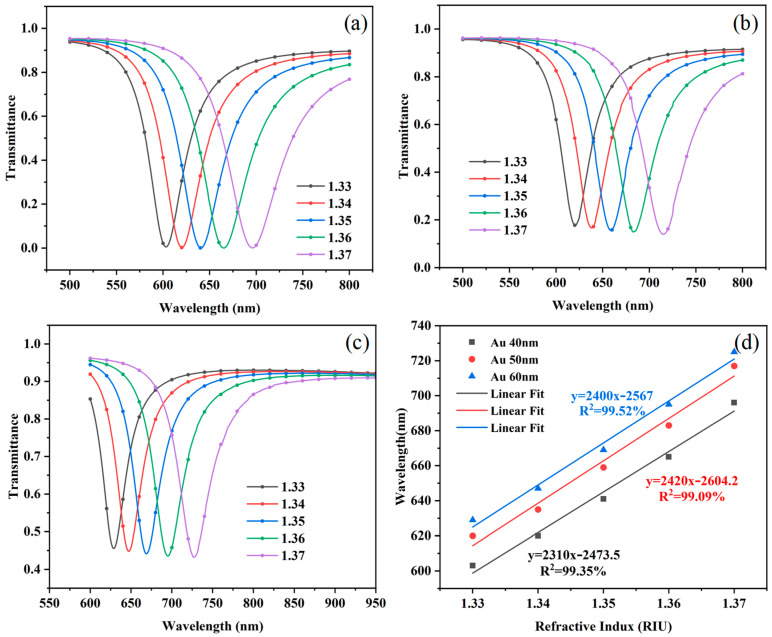
Simulation of optical fiber sensors modified with Au films of different thicknesses. (**a**–**c**) RS of Au film thicknesses of 40–60 nm. (**d**) Linear fitting results at RW. Definitions: resonance spectrum (RS); resonance wavelength (RW).

First, simulations were conducted on the optical fiber SPR structure with Au film thicknesses of 40 nm, 50 nm, and 60 nm. The simulated external refractive index (RI) was set to range from 1.33 to 1.37 in steps of 0.01. In COMSOL fiber bending modeling and dual-core diameter selection, one needs to first build a straight fiber geometry model; through the bending operation, to generate a structure with a bending radius R, define the core, cladding, and other materials’ refractive index, select the beam envelope or full-wave electromagnetic simulation of the physical field, set the input base mode field and the output PML boundaries, optionally coupled to the solid mechanics in the analysis of the effect of bending stress on the refractive index and the impact of mesh delimitation in the Encrypted core–cladding interface and bending inner region, and analyze the mode field distribution, transmission loss, and mode coupling by parametric scanning at R; and select a core diameter based on single-mode and multimode transmission characteristics and compare the loss difference, mode field offset, and sensitivity of the two under the same R. The single-mode loss near the critical radius is more sensitive to bending, and multimode higher-order mode leakage loss grows faster; these results are ultimately combined with the simulation results and the actual requirements. For the optimization of the diameter, the process can use a two-dimensional axisymmetric model to simplify the calculation and, through the experimental data, correct the parameters. Under different Au film thickness conditions, the total transmittance was accurately calculated, and the corresponding resonance spectra (RS) were plotted, as shown in Figure 3a–c. From Figure 3a–c, it can be observed that the overall red-shift in the RS occurs with the increase in the thickness of the Au film. The physical mechanism of this phenomenon lies in the fact that an increase in the thickness of the Au film leads to an increase in the effective length of the electronic oscillations and a consequent decrease in the resonance frequency according to the theory of surface plasmon excitations, which causes a red-shift in the RS. Through the linear fitting of the RW, the sensitivity of the SPR sensor can be quantitatively assessed, and thus, its performance can be evaluated. As shown in Figure 3d, the linear fitting results show that the linearity of the sensor remains consistent under different Au film thickness conditions. However, the sensitivity showed a tendency to increase and then decrease with the thickness of the Au film. The sensitivity of the sensor reaches a maximum value of 2420 nm/RIU when the Au film thickness is 50 nm. Despite the small increase in sensor sensitivity, this can improve detection capability and accuracy in multiple areas. This result indicates that the Au film thickness has a significant effect on the performance of the SPR sensor and that there exists an optimal thickness value that allows for maximum sensor sensitivity.

**Figure 4:**
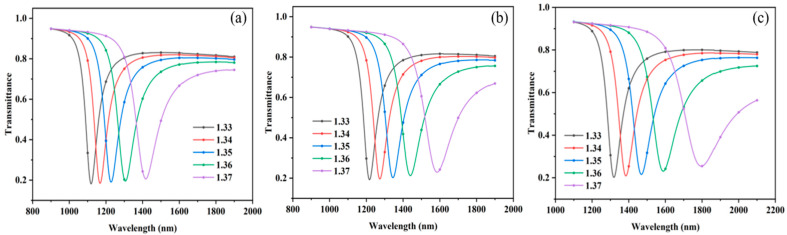
Simulation of optical fiber sensors modified with ITO films of different thicknesses. (**a**–**c**) RS of ITO film thicknesses of 60–80 nm.

**Figure 5:**
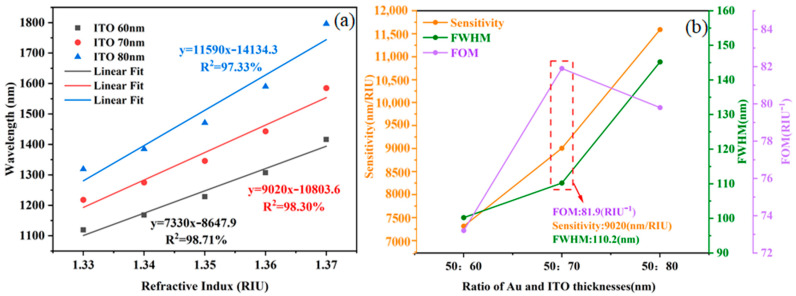
(**a**) Linear fitting results at the corresponding RW for ITO thicknesses of 60–80 nm. (**b**) Performance index of the optical fiber SPR sensing structure with different ITO film thicknesses.

Subsequently, further optimization of the ITO film layer structural parameters for the SPR sensor was carried out via simulation. With the thickness of the Au film fixed at 50 nm, the thickness of the ITO film was systematically adjusted to identify the optimal parameter combination. The RI of the external ambient medium was set within the range of 1.33–1.37. The total transmittance of the sensing structure was calculated for ITO film thicknesses of 60 nm, 70 nm, and 80 nm, and the corresponding resonance spectra (RS) were plotted, as illustrated in Figure 4a–c. The results show that the overall RS are red-shifted, and the FWHM gradually becomes wider as the thickness of the ITO film increases. Through the linear fitting of the RW, the sensitivity of the SPR sensor can be quantitatively assessed, and thus, its performance can be evaluated. The linear fitting results are shown in Figure 5a. In terms of linearity, the linearity deteriorates significantly when the ITO film thickness is 80 nm. In terms of sensitivity, the sensitivity of the sensor gradually increases with the increase in ITO film thickness, with a maximum sensitivity of up to 11,590 nm/RIU. This suggests that ITO film thickness has a significant effect on sensor performance and that there exists an optimal range of thicknesses that allows for maximum sensor sensitivity.

The performance metrics of the optical fiber SPR sensing structures with different ITO film thicknesses are demonstrated as shown in Figure 5b. The analysis shows that the FWHM of the sensor gradually increases with the increase in ITO film thickness. At an ITO thickness of 70 nm, the sensitivity of the sensor reaches 9020 nm/RIU, while the FOM reaches a maximum value of 81.9 RIU-1. Combining the three key performance indicators of sensitivity, FWHM, and FOM, the ITO film thickness of 70 nm performed the best in terms of performance and was therefore identified as the optimal structural parameter for the SPR sensor in this paper for subsequent sensor preparation.

## 3. Sensor Manufacturing and Experimental Setup

### 3.1. Materials

The materials used in the experiments included indium tin oxide (ITO) powder, chitosan (CS) powder, glacial acetic acid (CH_3_COOH, 99.8% purity), and polyacrylic acid (PAA) solution, all of which were purchased from Shanghai Aladdin Biochemical Technology Co (Shanghai, China). In addition, the multimode fiber (MMF in 62.5/125 μm) and single-mode fiber (SMF in 9/125 μm) were supplied by Wuhan Changfei Fiber Optic Cable Co. (Wuhan, China).

### 3.2. Conventional Au Film Sensor Manufacturing

The preparation of the fiber-optic/Au film sensor structure included two key steps: fusion splicing of the optical fiber and plating of the Au film. SMF and MMF provided by Wuhan Changfei Optical Fiber & Cable Co., Ltd. (Wuhan, China) were selected as the optical fiber substrate. The extent of the sensing area was determined by precisely controlling the length of the SMF, and the thickness of the Au film was controlled by adjusting the parameters of the metal film preparation apparatus. After the optimization experiments, it was found that the best sensor performance was achieved when the length of the sensing area was set to 1 cm and the coating time of the metal thin film apparatus was set to 90 s.

Firstly, fusion splicing of the sensing structure of the optical fiber sensor was performed. SMFs with a length of 1 cm were precisely intercepted using an optical fiber cutter to ensure that the end faces were flat. Subsequently, with the aid of a fiber fusion splicer, the two ends of the SMF were fused to a certain length of the MMF to form a multimode–single-mode–multimode structure. After fusion bonding was completed, the SMF section was burnished using an alcohol lamp to bend it into a U-shaped structure with a fixed bending diameter of 3 mm, a parameter that was optimized to ensure optimal sensor sensor performance [17]. Next, the prepared multimode–single-mode–multimode U-structured optical fiber sensor was fixed on a glass sheet and placed in an ion sputtering apparatus in preparation for plating the Au film. The thickness of the Au film was adjusted by controlling the coating time of the ion sputterer. In this experiment, the discharge duration was set to 90 s to prepare the Au film with a specific thickness. After completing the sputtering of the metal layer, the preparation of the optical fiber sensing region was completed.

### 3.3. Au/ITO Film Layer Structure Sensor Manufacturing

**Figure 6:**
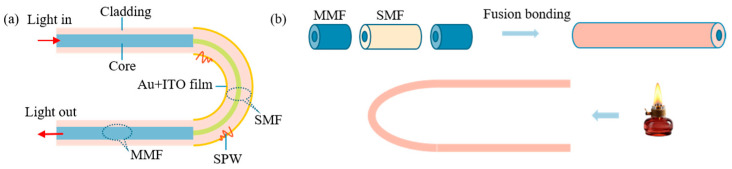
(**a**) Schematic structure of the U-shaped fiber SPR sensor. (**b**) Fabrication process of the U-shaped fiber SPR sensor.

Figure 6 shows the schematic structure of the U-shaped fiber SPR sensor and the fabrication process of the U-shaped fiber SPR sensor.

On the surface of the fiber/Au film structure, ITO film was further plated on its surface. Chitosan (CS) was chosen as an auxiliary coating material for its good film-forming and adsorption properties. In the experiment, ITO powder was dispersed in chitosan solution dissolved in CH_3_COOH to make the ITO surface positively charged. At the same time, the surface of the optical fiber/Au film was treated with a PAA solution to make the surface negatively charged. Based on the principle of positive and negative charge adsorption, the ITO film was plated on the surface of the Au film. Firstly, the PAA solution was uniformly coated onto the sensing area using a dip coater, with the coating process repeated for 50 cycles to ensure uniform negative surface charging. Subsequently, the treated ITO dispersion was coated onto the sensing region via dip-coating, with the number of coating cycles set to 10, 15, and 20, respectively. The film thickness increased with the number of cycles, resulting in ITO films of different thicknesses. Upon completion of the aforementioned steps, three Au/ITO optical fiber sensors with distinct ITO film thicknesses were successfully fabricated.

### 3.4. Performance Testing System

**Figure 7:**
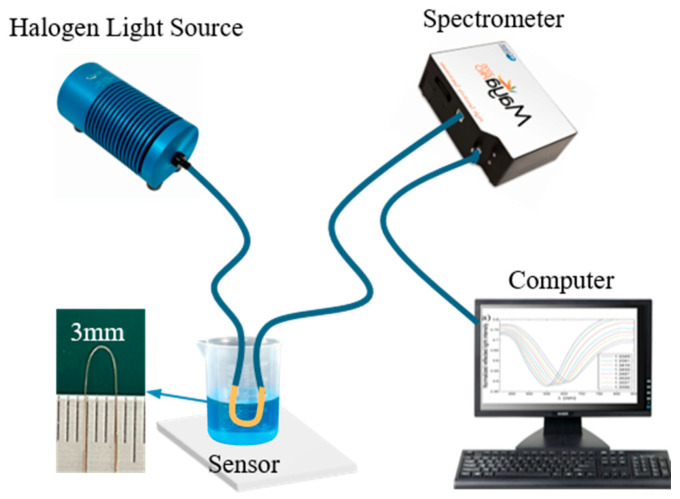
Schematic of sensor measurement.

The instrumentation used in the experiment included a halogen light source, a spectrometer, and a computer. During the experiment, the sensor was connected to the halogen light source (model DH-2000, produced by Ocean Optics (Shanghai, China)) and the spectrometer (model MAYA 2000 Pro, produced by Ocean Optics (Shanghai, China)) at its two ends, respectively. The spectrometer used had a resolution of 0.1 nm and a wavelength measurement range of 400–1100 nm. The spectrometer was connected to a computer via a data cable for data acquisition and analysis. In experimental operation, a solution with a specific RI is added to the surface of the sensor, then the sensor responds to the light signal from the halogen light source, which is detected by the spectrometer, and the corresponding transmission spectral information is transmitted to the computer and displayed in real time. The experimental measurement schematic is shown in Figure 7.

## 4. Results and Discussion

### 4.1. Refractive Index Sensitivity

When evaluating sensor performance, sensitivity (S) and figure of merit (FOM) are commonly assessed. Sensitivity (S) is defined as the ratio of the change in the resonance wavelength (Δλ) to the change in the refractive index (Δn) of the detected sample. Meanwhile, the figure of merit (FOM) is employed to provide a comprehensive evaluation of the sensor’s performance. Firstly, a series of sodium chloride solutions of different concentrations was prepared to simulate the external environment with different RIs. At a constant external ambient temperature, the concentration of a sodium chloride solution is linearly related to its RI, and it was therefore chosen to simulate the substance under test. The RI of the solution was measured accurately by adding the appropriate amount of the prepared sodium chloride solution dropwise to an Abbe refractometer. The RIs of the prepared solutions were tested to be 1.334, 1.343, 1.354, 1.364, and 1.374 RIU.

**Figure 8:**
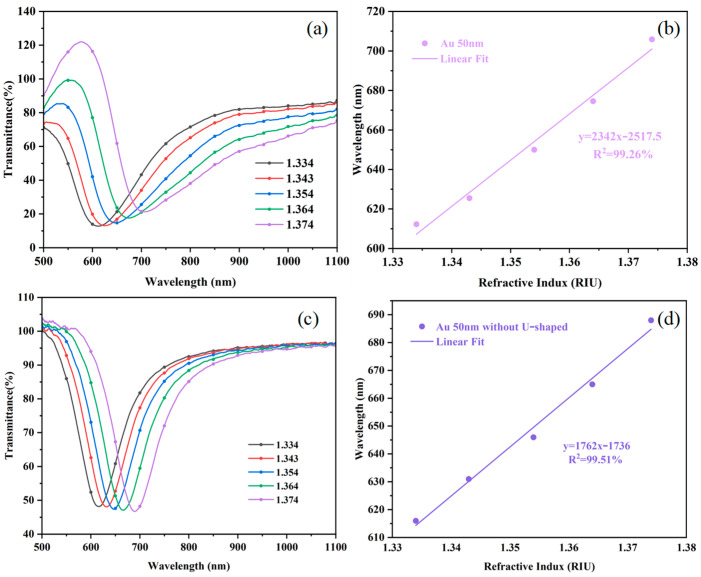
(**a**) RS at a 90 s time length of the plated Au film. (**b**) RW fitting plot. (**c**) RS at a 90 s time length of the plated Au film without the U-shape. (**d**) RW fitting plot.

The prepared Au film optical fiber sensor was used to measure the solutions with different RIs. Measurements were made on the formulated RI solutions, and the corresponding RS were obtained, as shown in Figure 8a. The lowest point in the spectrum corresponds to the RW at an RI of 1.334–1.374 RIU. A linear fitting to the RW was performed, and the results are shown in Figure 8b. The fitting result shows that the sensitivity of the sensor is 2342 nm/RIU and the linearity fitting result is 99.26%, indicating that the data meets the linear fitting condition. The FWHM of the RS was further calculated to be 181 nm. Combined with the sensitivity value, an FOM of 12.9 RIU-1 was obtained. This result shows that the prepared Au film optical fiber sensor has high sensitivity and good linearity in measuring the RI change, which is suitable for high-precision RI detection. The results for the structure without a U-shape are shown in Figure 8c,d. The sensitivity of the sensor is 1762 nm/RIU, which is a good indication that the U-shape structure effectively improves the sensitivity of the sensor.

**Figure 9:**
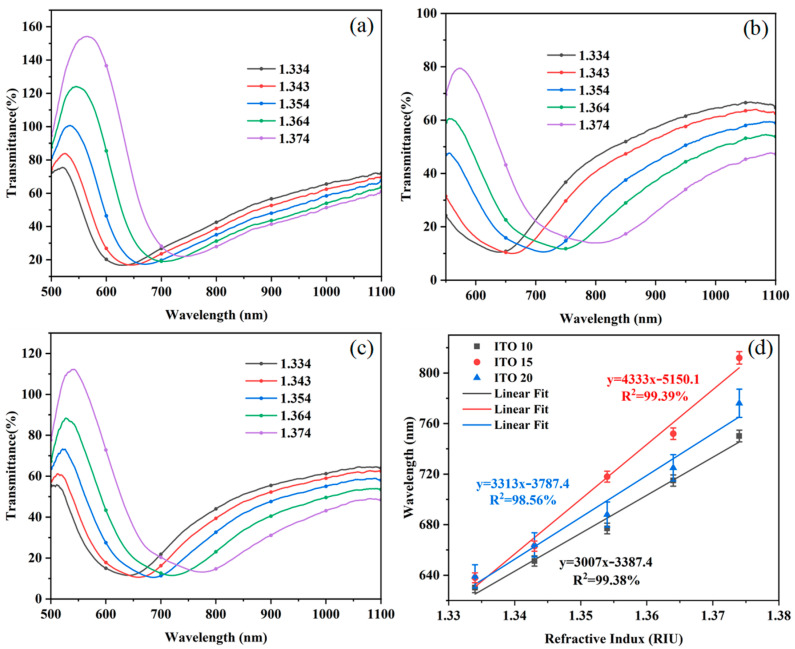
Transmission spectrum of Au/ITO film sensors. (**a**–**c**) RS for 10, 15, and 20 cycles of the coater. (**d**) RW fitting curve.

The three prepared Au/ITO optical fiber sensors with different ITO film thicknesses were used to detect the sodium chloride solutions with different RIs in turn, and the corresponding RS were obtained, as shown in Figure 9a–c. Compared with the conventional Au film optical fiber SPR structure, the sensor with the additional ITO film undergoes a significant red-shift in the RW, and the FWHM is significantly widened, which suggests that the introduction of the ITO film significantly enhances the performance of the sensor.

Through the fitting of the RW, the fitted curve is obtained, as shown in Figure 9d. Data integrity and scientific rigor are confirmed through error bars. The results show that the sensitivity of the sensor showed a tendency to increase and then decrease with the increase in the number of cycles of the dip coater. When the number of cycles of the dip coater is 20, the sensitivity of the sensor decreases instead, while the FWHM increases significantly. Therefore, considering the balance between sensitivity and full width at half maximum (FWHM), the optimal condition was ultimately determined to be 15 cycles of the dip coater, corresponding to an ITO film thickness of approximately 70 nm. This selection aligns with the simulation results in the earlier section regarding the influence of ITO layer thickness on performance. Under this condition, the sensitivity of the sensor reached 4333 nm/RIU, and the linearity fit result was 99.39%, which meets the linear fit requirement. By calculating the FWHM of the RS to be 199.5 nm and combining it with the sensitivity value, we obtained an FOM of 21.7 RIU^−1^. Compared to the conventional Au film SPR sensor, the sensitivity of the sensor after plating the ITO film 15 times was increased by 1991 nm/RIU, while the linearity was also improved, indicating a better fit.

**Figure 10:**
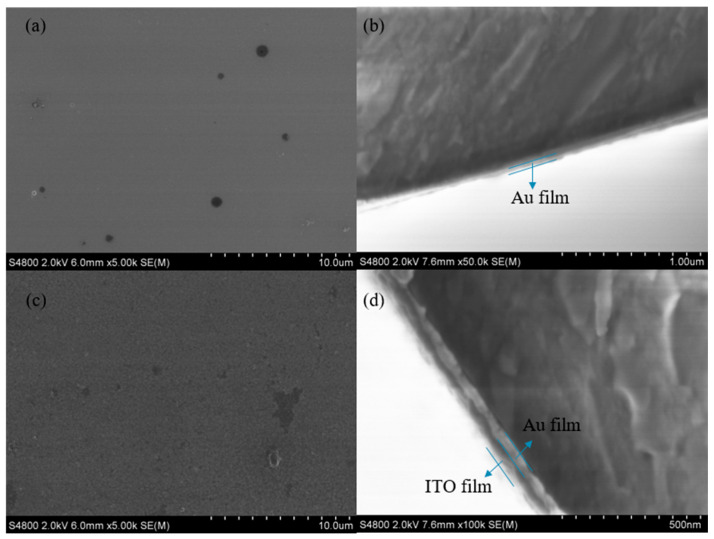
Scanning electron micrographs of the sensor. (**a**) Au film surface. (**b**) Fiber/Au film cross-section. (**c**) ITO film surface. (**d**) Fiber/Au film/ITO structure cross-section.

Figure 10 shows a scanning electron microscope (SEM) image of the sensor. The SEM used in this study has a measurement accuracy of 1 nm. From the SEM image, it can be clearly observed that the surfaces of the Au and ITO film layers are smooth and flat, without obvious breakage. The SEM images of the cross-section further show that the coating quality of the structure is good, with the Au and ITO film layers covering the fiber surface uniformly and tightly, with a Au film thickness of about 50 nm and an ITO film layer thickness of about 70 nm. The sensors fabricated in the experiments are often limited by the precision of the fabrication techniques and may have small dimensional deviations, surface roughness, irregular shapes, and other problems.

### 4.2. Verification of the Performance of Sensors Structured with Au/ITO Film Layers

**Figure 11:**
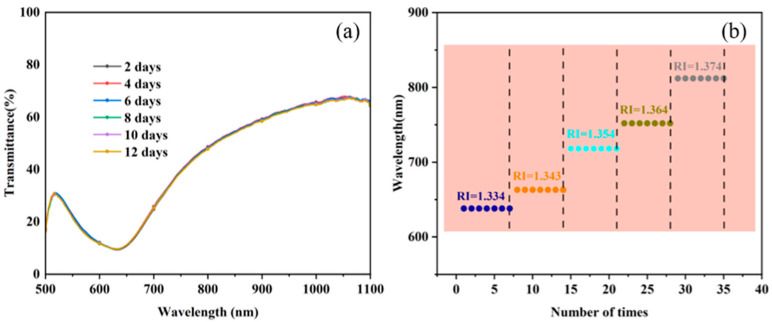
(**a**) RS of six repeatability tests. (**b**) RW of seven stability tests.

When testing RI solutions, the repeatability and stability of the sensor are key indicators to assess its performance. Therefore, the sensor was tested accordingly in this study. Six repetitive experiments were conducted with a 2-day interval when the RI of the surroundings was 1.334. The repeatability test results of the sensor with Au/ITO film layers are shown in Figure 11a. The results show that the RW remains almost constant over several measurements, exhibiting good consistency. The RSD was calculated to be 0.4236%, indicating the high repeatability of the sensor. This result demonstrates that the sensor exhibits stable performance in repeated measurements and can provide reliable support for the monitoring of complex biochemical reactions.

Stability is one of the key performance indicators of SPR sensors. In order to evaluate the stability of the proposed SPR sensor, in this study, it was placed in a solution with an RI of 1.334–1.374, and the change in the RW was continuously recorded, as shown in Figure 11b. The experimental results showed that the resonance peak remained relatively stable during the test without significant drift. Specifically, the maximum shift in the resonance peak was only 0.8 nm, corresponding to a maximum RI measurement error of 0.00018 RIU. This result shows that the proposed SPR sensor can maintain excellent stability during long-term monitoring and is suitable for high-precision detection in complex environments.

The performance of six different sensors was compared, and the results are summarized in Table 1. In the present study, the RI range of the substances measured was set at 1.33–1.37. As can be seen from Table 1, the SPR sensor proposed in this paper performs well in terms of sensitivity and has high detection performance. In addition, the sensor has a large FOM, which indicates a significant advantage in terms of detection accuracy.

## 5. Conclusions

In this paper, we found that materials with higher dielectric constants and conductivities can significantly enhance carrier motion efficiency and reduce energy consumption, thus enhancing the photocurrent signal strength and, thus, the EF strength and SPR effect, through a study of SPR sensors. Based on this principle, this paper further plated ITO film with a high dielectric constant and conductivity on the surface of traditional Au film to optimize the sensor design and detection performance. The experimental results show that the sensor proposed in this paper improves the EF strength by 1.12 times, sensitivity by 1.85 times, and the FOM by 1.68 times compared with the conventional Au film sensor. In addition, the sensor exhibits good repeatability and stability. The repeatability test results show that the RSD is only 0.4236%. The stability test results show that the maximum measurement error of the RI is 0.00018 RIU. This excellent performance makes this sensor have broad application prospects in the field of biochemical sensing.

## Figures and Tables

**Table 1:** Sensor performance comparison.

Sensor Structure	RI Range	S (nm/RIU)	FWHM (nm)	FOM (RIU^−1^)	Reference
Fiber/ELP Au film	1.333–1.344	2054	150	13.7	[18]
Fiber/Au film/MXene Ns	1.3343–1.3445	2431.4	275	8.8	[19]
MMF-HSF-MMF substrate	1.333–1.345	2000	130	15.4	[20]
Tapered fiber substrate	1.332–1.342	2266	120	18.9	[21]
D-shaped Au film/(Ti_3_C_2_Tx)	1.3335–1.3541	3143	206	15.3	[22]
U-shaped Au/ITO film	1.334–1.374	4333	199.5	21.7	This work

## Data Availability

Data is contained within the article. For further information or data requests, the authors are available and can be contacted.
